# Predictor Variables and Screening Protocol for Depressive and Anxiety Disorders in Cancer Outpatients

**DOI:** 10.1371/journal.pone.0149421

**Published:** 2016-03-08

**Authors:** Manuela Polidoro Lima, Adhemar Longatto-Filho, Flávia L. Osório

**Affiliations:** 1 Pio XII Foundation—Cancer Hospital of Barretos, Barretos, Brazil; 2 Laboratory of Medical Investigation (LIM-14), School of Medicine, University of Sao Paulo, Sao Paulo, Brazil; 3 Life and Health Sciences Research Institute (ICVS), School of Health Sciences, University of Minho, Braga, Portugal; 4 ICVS/3B’s—PT Government Associate Laboratory, Braga/Guimarães, Portugal; 5 São Paulo University, Medical School of Ribeirão Preto, São Paulo, Brazil; Technion—Israel Institute of Technology, ISRAEL

## Abstract

**Background:**

Cancer patients are at increased risk of persistent depressive and anxiety symptoms and disorders compared to the general population. However, these issues are not always identified, which may worsen the prognosis and increase morbidity and mortality. Therefore, the objectives of this study are to identify predictor variables (demographic and clinical) for the development of mood and anxiety disorders in cancer outpatients and to propose a probabilistic screening protocol considering these variables and certain standardized screening instruments.

**Methods:**

A total of 1,385 adults, of both genders, receiving outpatient cancer care were evaluated using a questionnaire and screening instruments. Thereafter, 400 of these subjects responded to the Structured Clinical Interview for the Diagnostic and Statistical Manual of Mental Disorders, Fourth Edition (SCID-IV) by telephone to confirm or rule out the presence of a Current Major Depressive Episode (CMDE) or Anxiety Disorder (AD).

**Results:**

Of the patients surveyed, 64% met the criteria for CMDE and 41% for AD. Female gender was found to be a risk factor for both disorders, and the presence of previous psychiatric history and marital status (divorced and widowed) were risk factors for anxiety disorders. When scoring above the recommended cutoff score, the screening instruments also indicated a risk of the studied disorders. Based on these findings, a screening protocol and nomograms were created for the quantification, combination and probabilistic estimate of risk, with accuracy indicators >0.68.

**Conclusion:**

The prevalence rates for the disorders under study are extremely high in cancer patients. The use of the proposed protocol and nomogram can facilitate rapid and wide screening, thus refining triage and supporting the establishment of criteria for referral to mental health professionals, so that patients can be properly diagnosed and treated.

## Introduction

Compared with the general population, cancer patients are at increased risk of persistent depressive and anxiety disorders and symptoms. These conditions may appear as a reaction to the diagnosis itself and/or throughout the treatment and rehabilitation processes [1] because cancer patients experience different types of loss and different levels of stress and emotional distress [2].

Likewise, the deterioration in physical appearance, disability and life changes associated with clinical disease are also risk factors for psychiatric disorders, particularly in individuals with a family history thereof [3]. There are also clinical conditions associated with cancer that can lead to the emergence of such symptoms [4,5].

Depression is the most common psychiatric disorder in cancer patients, with prevalence rates ranging from 13% to 40% [6,7]. This condition is associated with a worse prognosis for cancer treatment and increased morbidity and mortality [8]. Similarly, the prevalence of anxiety in cancer patients can vary between 10% and 30% [9,10].

Despite these high prevalence rates, there are many barriers to the identification and treatment of these disorders. Such barriers include the overlapping of emotional and clinical symptoms, the lack of preparation/training of healthcare professionals who are not specialized in mental health for evaluating emotional/psychological symptoms, the limited time for researching these matters, the difficulty involved in accessing specialized psychiatric care, and even the clinical and emotional distortion of the patients for critically evaluating their emotional state and seeking help [11].

Thus, establishing predictors of psychiatric disorders in cancer patients and being able to screen for them can facilitate their early recognition and treatment, which is necessary and important for better management of oncological diseases and for improving patients' quality of life [12].

The evaluation of these aspects has been recommended and implemented in clinical settings, in accordance with the recommendations of the International Psycho-Oncology Society [13], National Comprehensive Cancer Network [14] and American College of Surgeons Commission on Cancer [15, 16].

Screening instruments and protocols, especially those that are fast and easy to use and that consider such predictor variables, can be of great help and value, overcoming challenges related to cost and time and their use by healthcare professionals who are not specialized in mental health and do not have previous training.

Numerous mental disorder screening scales are available, some of which have been validated for use in cancer care. However, the sensitivity and specificity of such instruments are not always measured, and when they are, they do not always prove satisfactory (low sensitivity) [17]. Moreover, these scales are most often used in isolation, without considering other clinical and sociodemographic variables possibly associated with symptom onset. Thus, the use of these instruments does not meet the preconized recommendations for the Implementation of Distress Screening Programs in Cancer Centers [18] which, in general, points out the need of short, validated and multidimensionais instruments [19], Additionally, to our knowledge, there are no protocols available in the literature for mental disorders in cancer patients that offer a probabilistic estimate of the onset of said disorders, which would enable defining priorities for referral and treatment in specialized services. The available protocols, such as the one proposed by Pasquini et al [20], are extensive, and their use is limited to mental health specialists with previous training in diagnostic interviews. These characteristics difficult to implementation and the routine use of them in the different cancer patient care services [19,21].

Given the above, this study was developed to identify predictor variables (demographic and clinical) for the development of mood and anxiety disorders in cancer outpatients and to propose a probabilistic screening protocol, considering these variables and certain standardized screening instruments, that can be used in a quick manner and by different healthcare professionals who are not specialized in mental health.

## Materials and Methods

### Subjects

The sample in this study included 400 adult cancer outpatients from a specialized cancer hospital. The hospital is an outpatient public hospital at which approximately 3,800 new patients and 45,000 returning patients with differing types of cancer are treated per year. Initially, as part of a larger study [22], a total of 1,384 patients responded privately and individually to the screening instruments (exclusion criteria: severe cognitive impairment as qualitatively evaluated by the applicator, and the absence of clinical conditions that would affect responses to the instruments). Next, approximately one-third of the patients (N = 434) were selected to participate in a second data collection phase using a random number table. These patients were interviewed by telephone to obtain their response to the SCID-VI, and the presence or absence of a psychiatric diagnosis was confirmed. This step was conducted by professionals who were trained to administer the instrument. The calculated diagnostic consistency rate was greater than 85%. Overall, 34 subjects were not located (exclusion criteria), and the final sample consisted of 400 subjects. This sample is representative of the major sample (p>0.05).

### Instruments

The following screening instruments were used for data collection:

1. Patient Health Questionnaire-2 (PHQ-2): A self-administered instrument consisting of two items rated on a Likert scale of zero (never) to three (almost every day), which aims to screen for depression indicators [23]. The version validated for the Brazilian population [24], whose sensitivity and specificity were 1.00 and 0.98, respectively, was used. Recently, in the cancer context, this instrument showed sensitivity 0.53 and specificity of 0.88 for cutoff score ≥3. [17]
2. Generalized Anxiety Disorder (GAD-7): A self-administered instrument consisting of seven items, evaluated on a scale of zero to three, where zero means "never" and three means "almost every day". This instrument aims to screen for typical indicators of anxiety disorders experienced over the past two weeks [25]. This study used the version validated for the Brazilian population [26], with internal consistency and reliability values of 0.91. For the oncological context, GAD-7 presented for cutting ≥6 sensitivity of 0.52 and specificity of 0.77 [17].
3. Structured Clinical Interview for DSM-IV (SCID-IV—Clinical Version): An instrument used for establishing clinical psychiatric diagnoses based on the DSM-IV (Diagnostic and Statistical Manual of Mental Disorders, Fourth Edition). This instrument has been used as a “gold standard” in diagnostic validation studies among cancer patients in a range of settings [27,28]. It consists of a total of ten modules that can be applied independently or in combination according to the desired objective. Modules A (Mood Disorders) and F (Anxiety Disorders) were used for this study [29]. The version used was translated and validated for use in Brazil [30], and its reliability ranged from 0.61 to 0.90.
4. Sociodemographic and clinical identification questionnaire: This instrument was used to characterize the sample and encompasses the following variables: age, gender, education level, marital status, religion, work situation, previous history of psychiatric/psychological disorders, cancer care specialty, tumor location and stage, previous history of cancer treatment and of other clinical diseases. It is based on self-reported of the subject and in medical records.

### Data collection

As part of the larger study, patients were approached at the time of an outpatient medical visit and were asked to participate in the study by responding to the screening instruments. At this stage, the researchers provided a notebook with the instruments and remained available for any clarification and assistance needed. After completing the questionnaires, subjects were told they would receive a call to participate in the second phase of the study, at least seven and at most 14 days after the first stage of data collection. Using a random number table, 400 subjects were randomly selected for a telephone assessment using the SCID-IV, which was conducted by two properly trained mental health professionals who were blind to the subjects' scores on the instruments. The calculated diagnostic consistency rate was greater than 85%.

This study was approved by the local research ethics committee (Comitê de Ética em Pesquisa do Hospital de Câncer de Barretos- Fundação Pio XII; Process No. 537/2011), and all subjects provided written informed consent after being fully informed regarding the research procedure.

### Data analysis

The data were entered into a database by research auxiliary technicians, who were responsible for anonymizing the data. SPSS statistical software was used for the analyses.

The Chi-square and Student’s t tests were used to determine the association between sociodemographic and clinical characteristics with the screening scales applied in the first stage and with the disorders evaluated by the SCID-IV.

Subsequently, multiple logistic regression was used to assess the joint relationship of sociodemographic characteristics and scales with each SCID-IV disorder. For this purpose, only characteristics with p-values less than or equal to 0.20 in simple analysis (group comparison) were selected.

The significance level for all other analyses was set at 0.05.

Nomograms were created to quantify the combination of different risk factors and to develop a probabilistic estimate of the onset of the disorders studied. R software [31] was used for this analysis.

Finally, the internal validity of the proposed nomogram was determined using the following measures: Kolmogorov D-statistic, area under the receiver operating characteristic (ROC) curve, sensitivity, specificity, positive and negative predictive values, and accuracy. The confidence intervals of these measures were calculated via nonparametric bootstrap with 5000 replications, using R software.

## Results

The sample consisted of 400 subjects of both genders (women: N = 246; 61.5%), predominantly married (N = 273; 68.4%), with varying degrees of education (incomplete/complete elementary education: N = 224; 56%), not active in the labor market (N = 243; 60.9%) and affiliated with some type of religion (N = 311; 80.6%). These subjects were treated in different cancer specialties, the majority in Mastology (N = 70; 17.5%), Upper Digestive Tract (N = 57; 14.3%) and Head and Neck (N = 54; 13.5%).

Of these patients, 32.5% (N = 130) were stage T1, 80.6% (N = 323) N0 and 90.8% (N = 363) M0. In total, 34.1% (N = 137) had already undergone chemotherapy, 33.4% (N = 134) had undergone radiotherapy and 71.9% (N = 288) had undergone surgery.

Of the subjects evaluated, 64 (16%) met the criteria for a diagnosis of Mood Disorder (Current Major Depressive Episode—CMDE) and 166 (41%) for Anxiety Disorder (AD—Panic Disorder, and/or Obsessive Compulsive Disorder, and/or Posttraumatic Stress Disorder, and/or Generalized Anxiety Disorder and/or Phobias).

Initially, some analyses were performed for comparisons between groups, having as a parameter the presence/absence of CMDE and AD, as evaluated by the SCID-IV.

Table 1 presents the variables analyzed and their significance level for each group.

**Table 1:** Significance levels of the different variables analyzed for each clinical group (Current Major Depressive Episode and Anxiety Disorder) and control.

Variable	CMDE (n = 64) *vs*. Control (n = 336)	AD (n = 166) *vs*. Control (n = 234)
Gender	**p < 0.001**	**p < 0.001**
Education level	p = 0.420	p = 0.820
Marital status	**p = 0.111**	**p = 0.088**
Work situation	**p = 0.090**	**p = 0.151**
Religion	p = 0.970	p = 0.924
Previous psychiatric history	**p < 0.001**	**p = 0.001**
Family psychiatric history	**p = 0.088**	**p = 0.039**
Previous psychological care	**p = 0.004**	**p = 0.034**
PHQ-2 (cutoff 3)	**p < 0.001**	—-
GAD-07 (cutoff 10)	—-	**p < 0.001**

CMDE = Current major depressive episode; AD = Anxiety disorder; vs. = versus; PHQ-2 = Patient Health Questionnaire-2; GAD-07 = Generalized Anxiety Disorder

Variables with a significance level lower than 0.20 (highlighted in bold in Table 1) were then selected for each disorder, and a multivariate logistic regression was performed, taking this set of variables as the initial model. Considering the unsatisfactory results of the initial models, new models were tested by removing the variables with the lowest significance level one by one until the final model was reached (Tables 2 and 3).

**Table 2:** Final multivariate logistic regression model, with Current Major Depressive Episode as outcome variable.

Variable	Category	Odds ratio	CI < 95%		P value
			Lower limit	Upper limit	
Gender	Female	1 [ref]	-	-	**0.002**
	Male	0.294	0.136	0.634	
PHQ-2/cutoff 3	No	1 [ref]	-	-	**< 0.001**
	Yes	7.139	3.888	13.108	
Constant		0.149	-	-	**< 0.001**

PHQ-2 = Patient Health Questionnaire-2; [ref] = reference variable; CI = Confidence interval

**Table 3:** Final logistic regression model, with presence of Anxiety Disorder as outcome variable.

Variable	Category	Odds ratio	CI < 95%		P value
			Lower limit	Upper limit	
Gender	Female	1 [ref]	-	-	**< 0.001**
	Male	0.409	0.255	0.655	
Previous psychiatric history	No	1 [ref]	-	-	**0.028**
	Yes	2.520	1.105	5.749	
Marital status	Single	1 [ref]	-	-	**0.032**
	Married	1.462	0.810	2.638	0.207
	Divorced	3.565	1.370	9.278	**0.009**
	Widowed	2.797	1.009	7.749	**0.048**
GAD-07/cutoff 10	No	1 [ref]	-	-	**< 0.001**
	Yes	3.100	1.793	5.359	
Constant		0.470	-	-	**0.008**

GAD-7 = Generalized Anxiety Disorder; [ref] = reference variable; CI = confidence interval

As shown in Table 2, male gender is a protective factor against the development of depressive disorder, reducing the risk by 71%. A score equal to or greater than three on the PHQ-2 instrument is indicative of CMDE because it increases the odds of presenting the disorder by more than seven-fold, thus evidencing its high screening capacity.

According to Table 3, the male gender is also a protective factor against the development of anxiety disorders. Men are 59% less likely to develop these disorders than women.

The variables previous psychiatric history and marital status also appeared as risk variables; subjects with previous psychiatric history were 2.52 times more likely to develop some type of anxiety disorder than subjects without this history. Divorced and widowed subjects were 3.565 and 2.797 times more likely to develop some type of anxiety disorder than single participants.

A score of ≥ 10 on the GAD-07 questionnaire was also identified as a risk indicator, supporting the screening value of this instrument.

**Fig 1:**
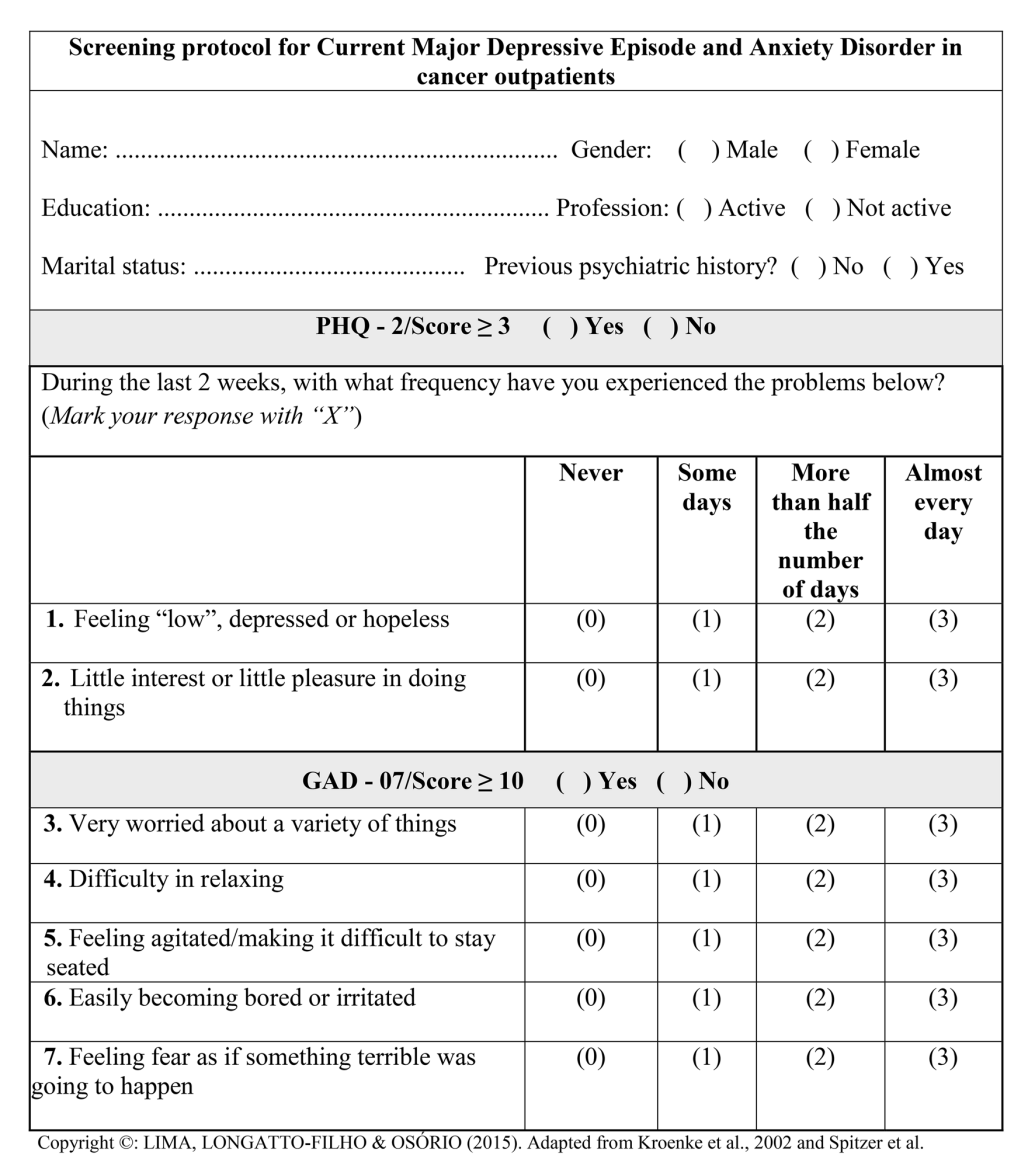
Screening protocol for Current Major Depressive Disorder and Anxiety Disorders in cancer outpatients.

**Fig 2:**
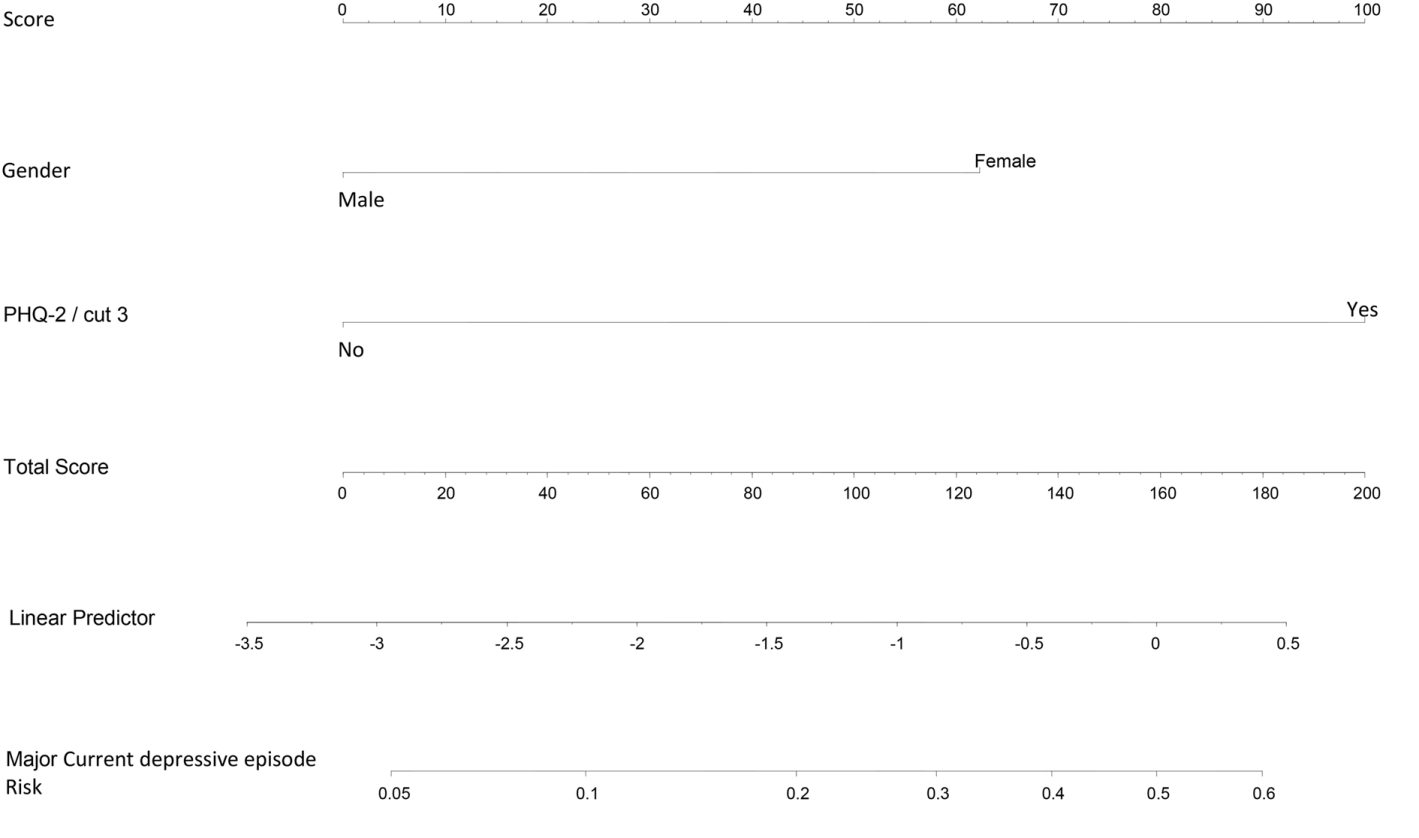
Nomogram: Risk factors for current depressive episode development.

**Fig 3:**
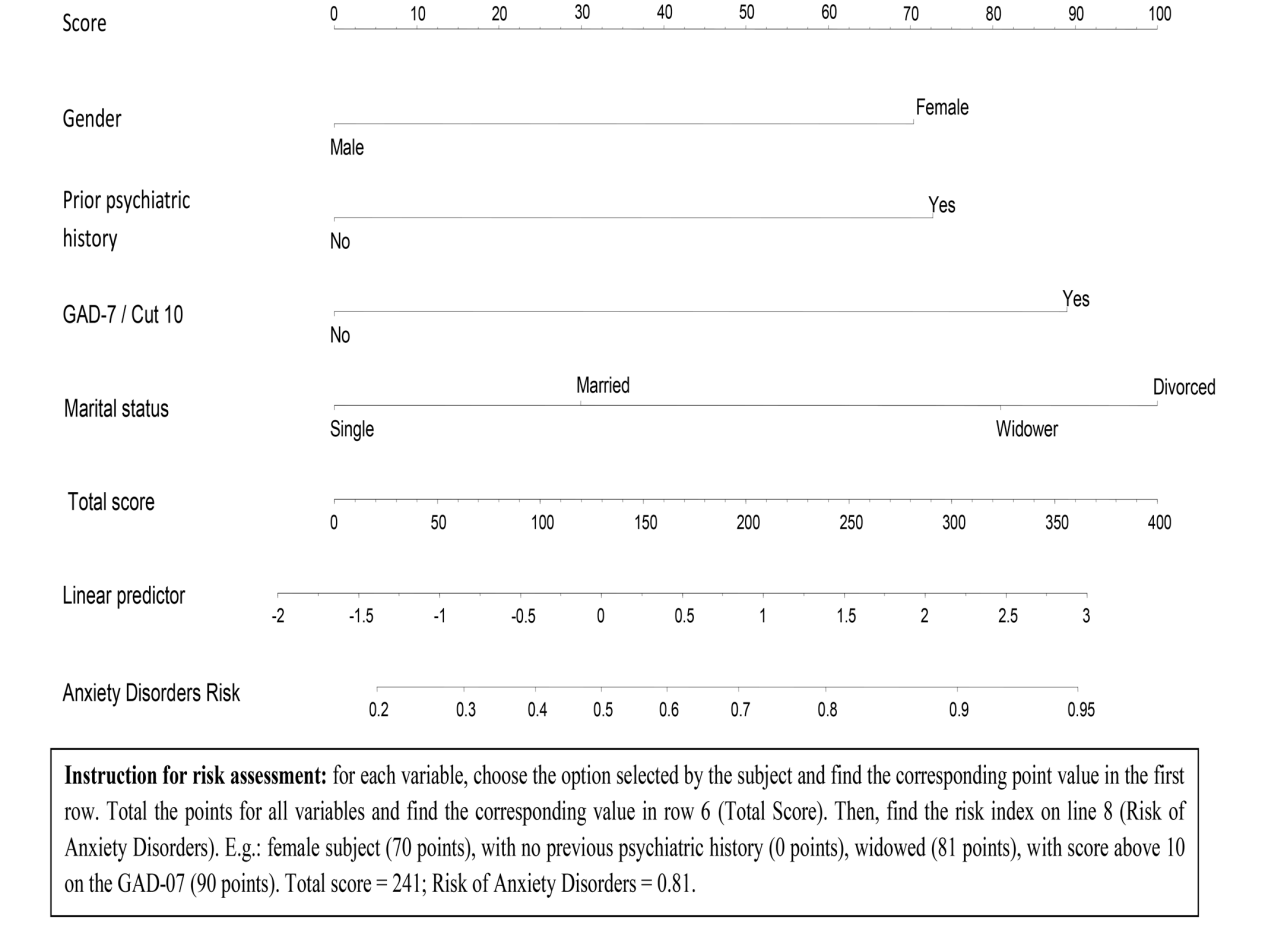
Nomogram: Risk factors for anxiety disorder development.

Considering all of the data presented, a specific protocol was created for the evaluation of these disorders. This protocol comprised five items and nomograms for risk assessment, which are shown in Figs 1–3.

The analysis of the internal validity of the nomograms indicated moderate sensitivity and high specificity, with accuracy values above 0.68. These data are detailed in Table 4.

**Table 4:** Indicators of Internal Validity of Nomograms.

	ANXIETY			DEPRESSION		
	Statistic	Confidence Interval -95% *		Statistic	Confidence Interval -95% *	
		Inferior	Superior		Inferior	Superior
D Statistic	0.316	0.248	0.409	0.415	0.309	0.547
PPV	0.656	0.567	0.734	0.466	0.354	0.582
NPV	0.693	0.636	0.743	0.908	0.875	0.939
Sensitivity	0.488	0.411	0.562	0.531	0.407	0.656
Specificity	0.819	0.762	0.861	0.884	0.85	0.918
Accuracy	0.682	0.633	0.722	0.828	0.79	0.863
ROC curve	0.711	0.659	0.761	0.771	0.711	0.829

* Via Bootstrap non-parametric

PPV = Positive predictive value; NPV = Negative predictive value

In S1 Fig, calibration of measures can be observed through the convergence graphics in which the measures bootstrap are concentrated around the original mean.

## Discussion

The present study showed high rates of CMDE and AD in the sample studied, and those rates are considerably higher than the prevalence rates for the general Brazilian population (11 and 19.9%, respectively) [32]. These results confirm previous findings in the literature that this population is at risk for such disorders, either because of their clinical condition or due to the whole spectrum of psychological conditions associated with the disease and coping with it, especially for diseases such as cancer, which are commonly associated with ideas of death [11].

The study results indicate the following predictor variables for the development of CMDE and AD: gender, previous psychiatric history, marital status, and score above the recommended cutoff in the screening instruments used.

With regard to gender, women had higher rates of depression and anxiety than men, indicating that being female is considered a risk factor for the development of such disorders. Previous studies that evaluated the role of gender in this context [33, 34] produced similar results, especially in the Gynecology and Mastology specialties [35, 36].

One possible explanation for these findings is that depression, considered a disease caused by genetic factors, stress and social pressure, is more common in women because they often experience work and responsibility overload due to their multiple duties, combining domestic work with childcare and often a professional career [37, 38]. This pressure becomes greater when facing illness and the inability to perform work activities, which can increase the risk factor, especially if combined with genetic/biological vulnerability, as studies also suggest that biological issues can be another possible explanation for the differences in depression and anxiety rates between the two genders [33, 39, 40].

Regarding the presence of previous psychiatric history, it is known that individuals with a history of psychiatric disorders, particularly if not treated properly, may be more susceptible to the onset of new disorders [41, 42]. Hereditary and constitutional factors, evaluated through family psychiatric history, may also influence the development of psychiatric disorders, which could explain the incidence of the same disorder in different members of a family. This feature can also be explained by the influence of the environment where these individuals live, as they share similar habits and are exposed to the same environmental and cultural factors [43, 44].

This variable had predictive value only for the development of anxiety disorders and not for depression. Nevertheless, in the group comparison, individuals with a previous psychiatric history also had higher rates of depressive symptoms, indicating its association with the onset of both symptoms and/or disorders, but with different weights.

The influence of the *marital status* variable on the onset of anxiety disorders was also identified in other studies. It is believed that the absence of a spouse and/or partner may increase the prevalence of anxiety and depression symptoms, as the help of the spouse and/or family and friends creates a support network that can help to strengthen the patient emotionally and help him or her adapt to the disease and treatment [45–47].

Regarding the instruments, the presence of a score above the cutoff value was also highly predictive of the onset of the studied disorders, which underlines the screening power of these instruments as shown in previous studies, including studies focusing on the cancer population [48, 49]. The easy, fast and wide-ranging use of these instruments may facilitate the identification of potential cases to be referred for evaluation by a specific professional and treated if necessary.

The proposed protocol was developed from the conjunction of predictor variables, which are associated with a nomogram providing a probabilistic estimate of the onset of disorders. It is believed that the use of this protocol, rather than exclusively utilizing the screening instruments, offers more reliable risk rates and allows greater refinement in triage, helping mostly by establishing referral criteria for mental health professionals. This difference is because it was observed that the sociodemographic and clinical characteristics cited can increase the risk for such symptoms/disorders by 15 to 40% by themselves.

Moreover, the integrated evaluation of the variables also enables establishing the priorities for referral and determining the best intervention, considering the specificity and weight of each risk factor.

This protocol can be used by any health professional in assisted applications or can be self-administered by the patient. Its use is free, and the estimated application time is two minutes. In the present study, the internal validity of this protocol, which was evaluated based on the discriminatory power of the nomograms, was determined to be promising. The values of sensitivity and specificity are good, and in according with others short instruments available in literature [18]. However, the practical applicability of this probabilistic protocol is being tested in a current study, and the findings (external validity) may further endorse its relevance, thus encouraging its use by cancer professionals and services. In addition, this protocol can be considered for other severe diseases that have depressive stigma similar to those of cancer and can serve as an optional armamentarium to appropriately interfere in the depression of the patients.

However, it must be noted that validated screening instruments and protocols are essential tools for overcoming the various barriers that prevent the appropriate identification and referral of cases to health care treatment. Nevertheless, such tools do not suffice to overcome other important challenges, such as a) the resistance of many patients who, despite being aware of their emotional condition, refuse help and treatment at this level [50], and b) the difficulty of access to and maintenance of specialized treatment, especially in underdeveloped countries such as Brazil [51], which requires the mobilization of efforts at different levels to raise awareness among these individuals and to improve health policies.

## Conclusions

The prevalence of the disorders under study is extremely high in cancer patients. The use of the proposed protocol and nomogram can facilitate rapid and wide screening, thus refining triage and supporting the establishment of criteria for referral to mental health professionals so that patients can be properly diagnosed and treated.

## Supporting Information

S1 Fig Calibration of measures through the convergence graphics (data, depression convergence, anxiety convergence)—this data set is fully anonymized (XLSX)

## Abbreviations

PHQ-2: Patient Health Questionnaire-2
GAD-7: Generalized Anxiety Disorder
SCID-IV: Structured Clinical Interview for DSM-IV
Current Major Depressive Episode—CMDE: Mood Disorder
AD—Panic Disorder, and/or Obsessive Compulsive Disorder, and/or Posttraumatic Stress Disorder, and/or Generalized Anxiety Disorder and/or Phobias: Anxiety Disorder
